# Seroprevalence Of SARS-COV-2 infection in asymptomatic indigenous from the largest Brazilian periurban area

**DOI:** 10.1371/journal.pone.0295211

**Published:** 2023-12-22

**Authors:** Laís Albuquerque de Oliveira, Marcelo dos Santos Barbosa, Alex José Leite Torres, Mariana Garcia Croda, Bruna Oliveira da Silva, Paulo César Pereira dos Santos, Regina Rossoni, Layla Oliveira Campos Leite Machado, Julio Croda, Crhistinne Cavalheiro Maymone Gonçalves, Michele Ferreira Marques, Tiago da Silva Ferreira, Silvia Inês Sardi, Gubio Soares Campos, Gabriel Barroso de Almeida, Marilia Maria Alves Gomes, Silvana Beutinger Marchioro, Simone Simionatto

**Affiliations:** 1 Health Science Research Laboratory, Federal University of Grande Dourados, Dourados, Mato Grosso do Sul, Brazil; 2 Laboratory of Immunology and Molecular Biology, Institute of Health Sciences, Federal University of Bahia, Salvador, Bahia, Brazil; 3 School of Medicine, Federal University of Mato Grosso do Sul, Campo Grande, Mato Grosso do Sul, Brazil; 4 Oswaldo Cruz Foundation, Campo Grande, Mato Grosso do Sul, Brazil; 5 State Secretariat of Health of Mato Grosso do Sul, Campo Grande, Mato Grosso do Sul, Brazil; Universitas Syiah Kuala, INDONESIA

## Abstract

This study assessed the seroprevalence of SARS-CoV-2 in 496 asymptomatic individuals from Mato Grosso do Sul, located in Dourados, the largest periurban indigenous area in Brazil, from January 25 to February 4, 2021. The volunteers participated before receiving their first dose of the CoronaVac inactivated vaccine. For screening, blood samples were collected and analyzed using SARS-CoV-2 rapid tests and the enzyme-linked immunosorbent assay (ELISA). We observed varying trends in total anti-SARS-CoV-2 antibodies across different variables. Seropositivity among the participants tested was 63.70% (316/496) using the rapid test and 52.82% (262/496) were positive using the ELISA method. The majority of participants identified with the Guarani-Kaiowá ethnic group, with 66.15% (217/328), and other ethnic groups with 58.84% (193/328). The median age of the subjects was 30.5 years, with 79.57% (261/328) being femaleThis research showed the elevated seroprevalence of SARS-CoV-2 antibodies in asymptomatic Brazilians. The findings indicate a high seropositivity rate among the asymptomatic indigenous population of Midwest Brazil. This underscores the overlooked status of these communities and underscores the need for targeted national initiatives that emphasize the protection of vulnerable ethnic groups in the fight against COVID-19.

## Introduction

The COVID-19 pandemic has accentuated the disparities faced by various population segments globally, especially in Brazil [1]. SARS-CoV-2 infection, similar to other infectious diseases, presents a higher risk to marginalized groups like the indigenous communities. This is due to a combination of personal, cultural, and economic reasons that render them especially susceptible, leading to disproportionate impacts [2]. Historically, these individuals face significant social, economic, and health challenges compared to the non-indigenous population, largely stemming from colonization and economic expansion [3]. Such challenges often hinder their ability to access health services [4]. In Mato Grosso do Sul (MS), the largest periurban indigenous region in Brazil, the close proximity of villages to urban hubs complicates the implementation of measures like social distancing. Furthermore, restricted access to clean water compromises regular hand hygiene, escalating their epidemiological risk [5].

Despite Brazil having a dedicated health system for its indigenous residents, which emphasizes primary health care within indigenous territories, there exist disparities in the COVID-19 data. Questions arise about the authenticity of the official figures reflecting the true impact of COVID-19 on this community, highlighting the importance of data integration from diverse sources, including entities operating within these territories [6]. The potential for asymptomatic transmission further complicates the COVID-19 landscape, especially in regions lacking prompt detection mechanisms [7].

Situated in Brazil’s Midwest, the state of Mato Grosso do Sul is home to the country’s second-largest indigenous demographic, totaling 83,434 individuals. Dominant ethnic groups include the Guarani-Kaiowá, Terena, and Guarani-Nhandeva, who collectively represent 96% of the state’s indigenous populace. Dourados-MS encompasses the country’s largest periurban indigenous area, spanning 3,474.50 ha, and shelters 18,000 inhabitants. Of this number, 16,400 (or 91.11%) reside in the Bororó and Jaguapirú villages [8]. Given the ongoing circumstances surrounding the COVID-19 pandemic, this study embarked on examining the seroprevalence of SARS-CoV-2, particularly focusing on paucisymptomatic infections, and discerning the determinants linked with COVID-19 among the asymptomatic indigenous residents of Dourados-MS.

## Materials and methods

### Environment and study population

**Fig 1:**
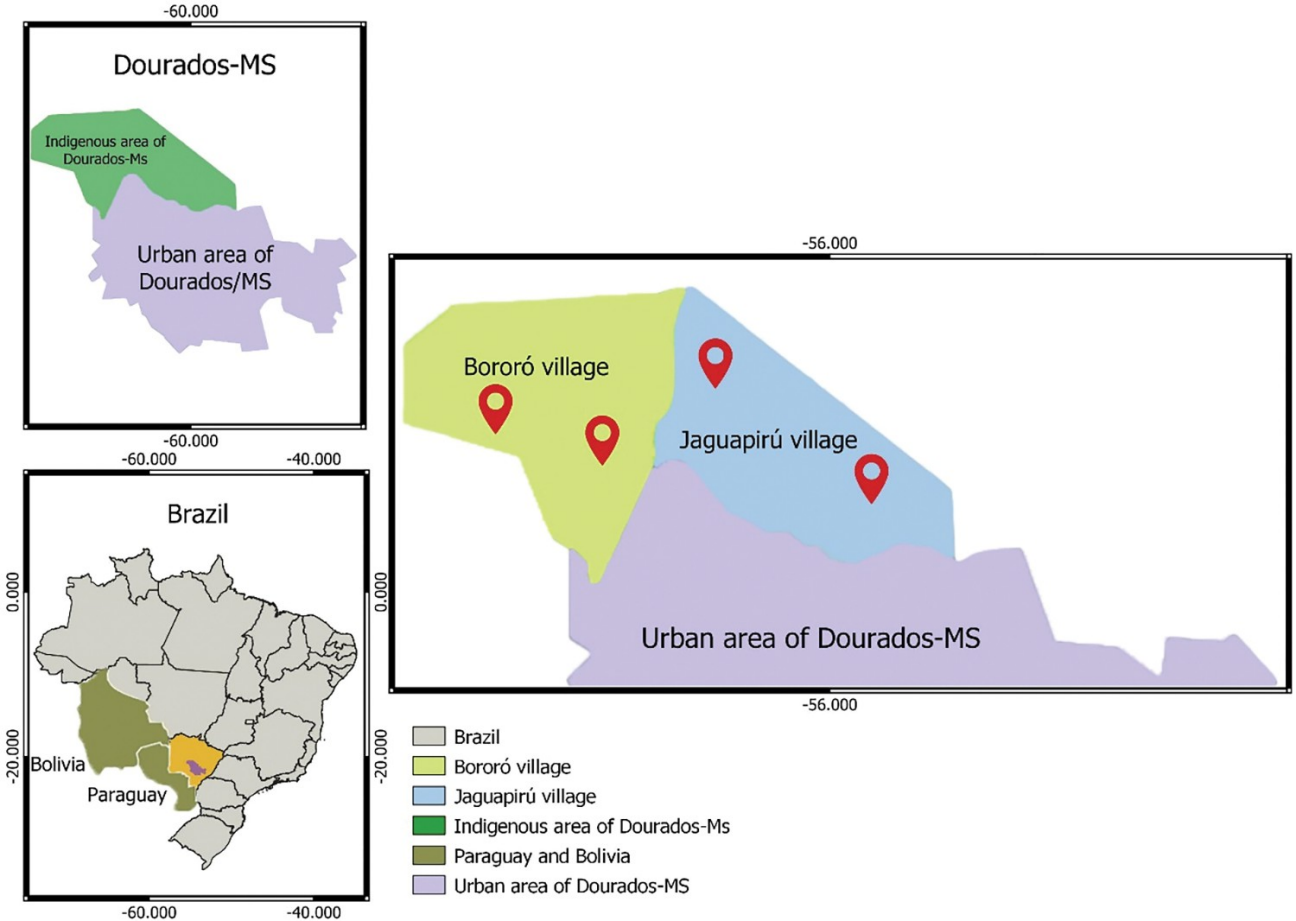
Map of the central Brazil region displaying the geographic positions of the Bororó and Jaguapiru villages in the city of Dourados, Mato Grosso do Sul state (MS). The map was created using the software QGIS 3.16, QGIS Geographic Information System (version 3.28.2 - https://www.qgis.org/).

This cross-sectional investigation focused on individuals from the indigenous community of Dourados-MS, located in the Midwestern region of Brazil. The study took place from January 25 to February 4, 2021. Participants were adults aged 18 years or older and had given their written informed consent. The study included healthy volunteers who displayed no symptoms related to COVID-19 and had not been vaccinated against the virus [9]. For the purpose of sampling and randomization, a cluster method was employed. The clusters were defined based on the primary health care unit’s location and were segmented into microregions in relation to the population they served. Consequently, the villages were categorized into four clusters, each encompassing a primary health care unit. Indigenous individuals invited to join the study were selected in a manner that ensured a balanced representation from each region, thereby ensuring a diverse participant pool from each village (Fig 1). The sample size determination was anchored on a total population of 18,000 indigenous inhabitants in Dourados-MS. The sample size was established using a projected SARS-CoV-2 prevalence of 50% in the Brazilian demographic, a 95% confidence interval (CI), and a precision of 0.5%. Additionally, 30% of participants were included to account for potential study dropouts.

### Data and blood collection

The research was segmented into two stages and employed the subsequent data collection methods: (1) Blood samples were obtained for SARS-CoV-2 serological tests. After ensuring antiseptic precautions, 4.5 mL of peripheral venous blood was drawn utilizing a vacuum tube method. These samples were then treated to separate the serum and subsequently stored at –20°C. (2) An interview was conducted using a self-report questionnaire which included into demographic details and aspects connected to COVID-19. Furthermore, participants were questioned on COVID-19 symptoms in the period prior to enrollment.

### Serological test

Blood samples collected were transported to the Health Sciences Research Laboratory at the Federal University of Grande Dourados where they were tested to ascertain the SARS-CoV-2 serological status. For this, the Leccurate COVID-19 rapid antibody test (Lepu Medical Technology Leccurate SARS-CoV-2 Antibody rapid test kit, China; Lot: 20CGG2511X Colloidal Gold Immunochromatography) was employed. This test detects IgM and IgG antibodies against SARS-CoV-2, with a sensitivity and specificity of 98.9% and 97.6%, respectively. According to the manufacturer’s protocol, 10 μL of whole blood was added to 100 μL of diluent and the test was interpreted 15 minutes after the reaction. Subsequent to the rapid test, samples underwent testing using two distinct enzyme-linked immunosorbent assays (ELISA): Anti-SARS-CoV-2 ELISA (IgG) (EI2606-9601 G) and Anti-SARS-CoV-2 NCP ELISA (IgM) (EI2606-9601-2 M). The reagent wells in these tests are coated with the modified nucleocapsid protein (NCP) of SARS-CoV-2 to respectively detect IgG and IgM antibodies against the virus. Both tests were administered following the manufacturer’s guidelines (EUROIMMUM, Germany). These tests operate on the principle of an indirect semi-quantitative ELISA. Patient samples were diluted at 1:100 (10 μL of the sample to 1 mL of sample buffer provided in the kit). The optical density readings were taken at 450 nm using a spectrophotometer (Multiskan FC, Thermo Scientific, USA). ELISA test results were determined based on the cutoff formula prescribed by the manufacturer. Cutoff values were derived by computing the ratio of the optical density (OD) values from either the control or the patient sample to the calibrator’s OD. The formula employed was “OD of control or patient sample/OD of the calibrator = ratio”. Ratios below 0.8 were labeled as negative, while those 1.1 or above were termed positive. Ratios between 0.8 and below 1.1 were deemed borderline, these samples with borderline values were retested in good time to ensure a reliable result, after which the results were classified as positive or negative. Every participant was personally apprised of their serological findings, and newly detected COVID-19 cases from this study were documented in the Notifiable Diseases database.

### Statistical analysis

The collected questionnaire responses and serological test results were thoroughly consolidated and verified as part of a quality assurance process, after which they were input into the Research Electronic Data Capture software, a freely available platform [10]. Descriptive statistics were employed to detail the population’s characteristics, with findings conveyed as percentages (%). Pearson’s chi-square (χ^2^) test or Fisher’s exact test was applied to discern any significant disparities between categorical variables. Univariate Odds ratios, complemented by 95% confidence intervals (95% CI), were derived from contingency tables to establish associations. A p-value < 0.05 was considered for statistical significance. Data analysis was executed using the IBM SPSS Statistics 2.0 software (Chicago, USA).

### Ethical approval and consent to participate

The study involving human participants was reviewed and approved by the Brazilian Research Ethics Committee (CONEP), an ethics Committee on Human Research, protocol number 4.502.250. The participants provided their written informed consent to participate in this study, this was done via an interview presenting the objectives of the research and answering the volunteers’ doubts, all those who agreed to participate received the free and informed consent form (TCLE) to sign their consent. All experiments were conducted following the relevant guidelines and regulations following the guidelines set forth by CONEP. Confidentiality and the right to leave the study at any time were guaranteed to all participants.

## Results

### Sociodemographic characteristics

**Fig 2:**
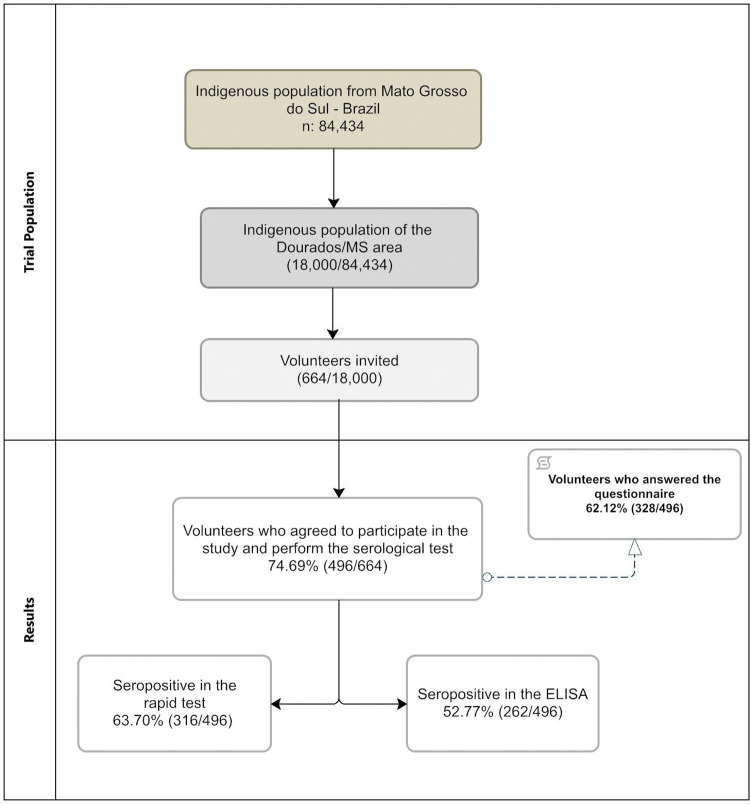
Flowchart of the screening and design of the study.

Out of the 664 healthy volunteers who were approached, 74.69% (496/664) agreed to take part in the study. Blood samples were collected from these participants to conduct serological tests, including rapid tests and ELISA. Among the participants, 66.12% (328/496) were willing to respond to the self-reported questionnaire (Fig 2).

The participants’ average age was 30.5 years. Among the participants, 52.62% (261/496) were women, 58.84% (193/328) were from the Bororó village, and 66.15% (217/328) belonged to the Guarani-Kaiowá ethnicity. Regarding education, 86.28% (283/328) of participants had received 5–8 years of regular schooling. Additionally, 46.65% (153/328) reported an income below 200 USD (below the Brazilian minimum wage), and 77.13% (253/328) received some financial assistance from the government. Moreover, 81.10% (266/328) did not have a stable job or formal income. Regarding living conditions, 90.55% (297/328) had access to water supply, and 39.33% (129/328) lived in residences with up to five individuals. Furthermore, 34.76% (114/328) reported accessing official information about COVID-19 through more than one means of communication, such as radio, television, cell phones, or the internet (Table 1).

**Table 1:** Seroprevalence of SARS-CoV-2 rapid testing according to sociodemographic characteristics.

Variable	n (328)	%	Rapid Test +	%	OR	CI 95%	*P*
**Age**							
18–20	78	23.78	43	55.13	0.788	0.466–1.301	0.340
21–40	193	58.84	122	63.21	1.416	0.905–2.216	0.127
41–60	46	14.02	24	52.17	0.697	0.373–1.304	0.259
61–90	11	3.35	7	63.64	1.185	0.340–4.131	0.789
**Gender**							
Female	261	79.57	151	57.85	0.671	0.381–1.182	0.167
Male	67	20.42	45	67.16	1.490	0.846–2.624	
**Village**							
Bororó	193	58.84	124	62.24	1.572	1.004–2.462	0.047
Jaguapirú	135	41.15	72	53.33	0.635	0.406–0.995	
**Ethnicity**							
Guarani-Kaiowa	217	66.15	135	62.21	1.349	0.848–2.145	0.205
Others	111	33.84	61	54.95	0.741	0.466–1.178	
**Marital status**							
Married	230	70.12	132	57.39	0.715	0.437–1.169	0.181
Unmarried	98	29.88	64	65.31	1.397	0.855–2.284	
**Work outside village**							
No	266	81.10	157	59.02	0.849	0.480–1.502	0.574
Yes	62	18.90	39	62.90	1.177	0.665–2.082	
**Familiar monthly incomes (USD)**							
< 200	153	46.65	88	57.52	0.839	0.539–1.307	0.439
200–488	157	47.87	97	61.78			
> 488	18	5.49	11	61.11			
**Government benefit**							
Yes	253	77.13	149	58.89	0.853	0.502–1.451	0.558
No	75	22.87	47	62.67	1172	0.689–1.992	
**Education level**							
least 4 years of study	45	13.72	29	64.44	1.259	0.654–2.423	0.490
5 to 8 years of study	283	86.28	167	59.01	0.794	0.412–1.528	
**People living in the house**							
1 resident	17	5.18	14	82.35	3.307	0.931–11.745	0.06
2 residents	31	9.45	17	54.84	0.8	0.380–1.685	0.586
3 residents	71	21.65	44	61.97	1.125	0.656–1.931	0.667
4 residents	81	24.70	49	60.49	1.041	0.623–1.739	0.876
5 or more residents	129	39.33	72	55.81	0.764	0.486–1.198	0.241
**Access to piped water**							
Yes	297	90.55	176	59.26	0.8	0.369–1.730	0.570
No	31	9.45	20	64.52	1.25	0.578–2.703	
**COVID-19 information sources**							
Cell phone	214	65.24	91	42.52	1.317	0.824–2.105	0.249
Others	114	34.76	41	35.96	0.759	0.475–1.213	
**Other risk behaviors**							
**Vaccinated influenza**							
Yes	290	88.41	167	57.59	0.421	0.192–0.922	0.030
No	38	11.59	29	76.32	2.373	1.084–5.194	
**Contact with COVID-19 suspect**							
Yes	218	66.46	124	56.88	0.696	0.432–1.120	0.135
No	110	33.54	72	65.45	1.436	0.892–2.311	

*The proportion used to infer the seroprevalence was proportion of positive results for rapid test. CI Confidence Interval. p < 0.05. Regardless of quantity. (+) Positive results in rapid test. The results compiled in this table represent the volunteers who answered the self-reported questionnaire.

### Seroprevalence of SARS-CoV-2 antibodies

The highest percentage of positivity was observed among individuals who live alone (82.35% [14/17]), those not vaccinated for influenza (76.32% [29/38]), unmarried individuals (65.31% [64/98]), those without access to piped water (64.52% [20/31]), those with less than 4 years of formal schooling, and those who work outside the village (62.90% [39/62]). Regarding the rapid test, 63.70% (316/496) of the volunteers showed seropositivity for SARS-CoV-2 antibodies, with 24.19% (122/496) testing positive for IgM–IgG, 33.06% (164/496) for IgM only, and 6.04% (30/496) for IgG. On the other hand, using the ELISA test, 52.77% (262/496) of the volunteers were seropositive for SARS-CoV-2 antibodies, with 20.56% (102/496) testing positive for IgM–IgG, 20.96% (104/496) for IgM, and 11.29% (56/496) for IgG (Table 2).

**Table 2:** Seropositivity of serological tests grouped by immunoglobulin detected according to the proportions of the results.

Antibodies detected	Rapid Test (n = 496)	%	ELISA (n = 496)	%	OR	IC 95%	*P*
IgM+	164	33.06	104	20.96	2.05	1.498 - 2.804	0.001
IgM/IgG +	122	24.60	102	20.56	1.55	1.121 - 2.156	0.008
IgG +	30	6.04	56	11.29	0.69	1.121 - 2.156	0.143
Total positives	316	63.70	262	52.77	1.56	1.216 - 2.021	0.000
Total negatives	180	36.30	234	47.20	0.63	0.494 - 0.822	0.000
Predictive value*							
Positive	-	79.74	-	96.18	-	-	-
Negative	-	94.44	-	72.64	-	-	-

*Predictive value of positive and negative serology test results. (+) positive results.

Additionally, the ELISA test yielded 1.08% (5/496) indeterminate results, and 47.20% (234/496) tested negative for any antibody. The concordance of seropositivity between the two tests was 50.8% (252/496) (S1 Table).

Among the asymptomatic volunteers (66.46% [218/328]), approximately two-thirds reported having had contact with COVID-19-positive individuals at some point in their lives [95% CI, 0.696 (0.433–1.120)]. Among these volunteers, 56.90% (124/328) tested positive on the rapid serological test. Specifically, 54.83% (68/124) tested positive for IgM, 33.06% (41/124) for IgM–IgG, and 12.09% (15/124) for IgG. On the other hand, among the 33.53% (110/328) of volunteers who had not been in contact with COVID-19-positive individuals, 65.45% (72/110) tested positive on serological testing. Out of these, 51.38% (37/72) were positive for IgM, 40.27% (29/72) for IgM–IgG, and 8.33% (6/72) for IgG.

**Fig 3:**
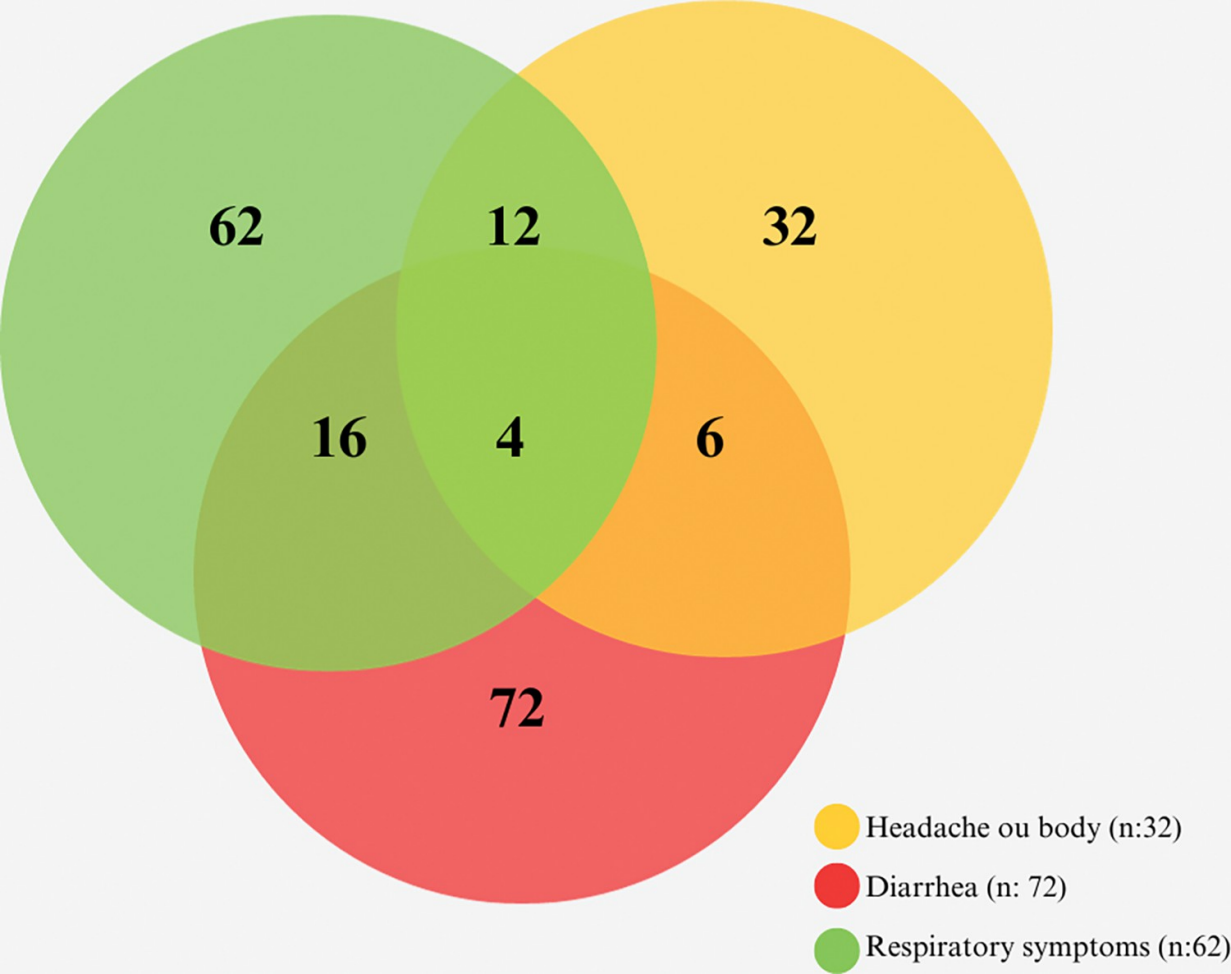
Venn diagrams illustrating reported symptoms related to COVID-19 disease. Volunteers reported all symptoms mentioned before undergoing testing for this study.

During the interviews, 50.60% (166/328) of the subjects reported having experienced symptoms related to COVID-19 before participating in our study. Among them, 19.27% (32/166) reported headaches or body aches, 37.34% (62/166) respiratory symptoms, 43.37% (72/166) diarrhea, and 2.40% (4/166) reported experiencing all these symptoms (Fig 3).

## Discussion

In this study, we screened asymptomatic indigenous individuals and found a high seroprevalence of SARS-CoV-2 antibodies (63.70%) within an indigenous community in MS, Brazil. Similar seroprevalence rates have been reported among symptomatic indigenous communities in Brazil [11, 12]. A separate screening study of Brazilians revealed that the seroprevalence among indigenous people was 4.71 (3.65; 6.08) times higher compared to the non-indigenous population [12]. Additionally, studies have indicated that indigenous populations residing closer to rural areas are disproportionately affected by COVID-19 [13, 14].

Studies conducted in the Amazon region of Brazil and a community in the Colombian Amazon revealed high seroprevalence rates for SARS-CoV-2 among native populations, with percentages ranging from 57.6% to 68.1% [15, 16]. Even among urban indigenous populations in Manaus, Brazil, a significant 60.71% showed seropositivity for the virus, despite the absence of reported COVID-19 symptoms [17]. These findings underscore the widespread impact of SARS-CoV-2 infection on indigenous communities, particularly in the Amazon region of Brazil [18].

Our study highlights the unexpectedly high exposure of asymptomatic indigenous individuals to the virus. Notably, the highest positivity was observed among those who live alone, are not vaccinated for influenza, are unmarried, lack access to piped water, have chronic diseases, have less than 4 years of formal schooling, and work outside the village. Vulnerability to the disease among indigenous individuals may be linked to their knowledge and behavior. Observational studies have indicated that indigenous people have less understanding about SARS-CoV-2, its transmission, preventive measures, and risk behavior than the general population [11, 13]. Consequently, several social and socioeconomic factors may contribute to the elevated seroprevalence observed in our study, and these should be taken into account to implement more effective disease control strategies. Considering the results presented and the reactive samples, it is possible to assess that asymptomatic individuals may have had contact with confirmed cases but were not evaluated during the exposure period. These undocumented infections often present mild symptoms, which go unnoticed, and depending on their contagiousness and numbers, they can expose a much larger portion of the population to the virus than what would typically be observed.

According to the released epidemiological notification data [19] only 59 patients with suspected COVID-19 or contacts with confirmed cases during the study period sought health services. Among them, 50.84% (30/59) tested positive through antigen testing or RT-PCR [20]. These positive cases represent only 0.32% (59/18,000) of the total indigenous population in Dourados-MS. However, a worrisome discrepancy emerges when comparing these data to the number of individuals tested in our study. The positive cases among asymptomatic individuals account for 63.7% (316/496) of the studied group or 1.72% (316/18,000) of the total population. This suggests a possible discrepancy between the number of confirmed cases and the actual number of infected individuals. Official data on COVID-19 cases and deaths in the indigenous population are available through the Information System for Indigenous Health Care. However, limitations in the quality, comparability, complementarity, timeliness, and transparency of these data may hinder a more reliable analysis of the COVID-19 scenario in the national indigenous population [11, 20]. Delays in reporting may result in increased incidence and mortality rates of COVID-19. Following WHO guidelines, controlling COVID-19 requires effective epidemiological surveillance among residents of indigenous communities and the health professionals serving them, including the isolation of suspected individuals and potential positive contacts, as well as periodic testing to identify symptomatic and asymptomatic individuals [19, 20].

In accordance with the guidelines of the national health surveillance agency (ANVISA) [21], molecular tests such as RT-PCR should be prioritized for detecting infections in symptomatic patients, those with mild symptoms, outpatient cases, and contacts of confirmed patients, especially in locations where molecular testing is limited, unavailable, or has long response times. However, using these tests to search for asymptomatic cases is not recommended. In our study, we employed rapid tests to screen and determine the seropositivity among asymptomatic individuals, aiming to identify the extent of their exposure to SARS-CoV-2. To ensure the reliability of the data, we confirmed the results using ELISA. The outcomes obtained from the rapid test and ELISA were highly similar, except for the IgM antibodies’ positivity. The notable percentage of individuals testing positive for IgM antibodies in our study might be attributed to the emergence of the *Gamma* variant’s circulation in Brazil [22], possibly causing a higher number of people with recent infections, which are detected by IgM positivity.

A comparison of the performance of seven different rapid tests with two Euroimmun ELISA demonstrated significant variations in IgM results between the tests [23]. This might be related to the fact that more patients experienced earlier seroconversion for IgG than for IgM [24]. Additionally, prior studies have described 100% seroconversion for IgG antibodies but not for IgM [25]. Immunoglobulin M is indicative of the acute phase of diseases, as it is the first antibody released when an infectious agent is encountered, and it acts as an activator of the immune system. On the other hand, Immunoglobulin G, due to its specificity, indicates the late phase of infection or previous contact with the infectious agent, serving as a memory antibody. The simultaneous presence of both IgM and IgG immunoglobulins suggests a recent infection, or the infected organism is transitioning to the late stage of the disease and may have developed immunity against the antigen [26]. The variation in IgM results between the two tests could be attributed to the rapid test’s tendency to provide false-positive results [27]. Thus, ELISA, a widely used method for clinical diagnosis with high specificity, is preferable for testing large-scale samples [13, 23, 28]. Despite these differences, the rapid test remains useful for monitoring the spread of SARS-CoV-2, especially for populations facing challenges in accessing basic healthcare facilities. It can be employed in various settings and is cost-effective [29].

This cross-sectional study has certain limitations. Firstly, data collection relied on questionnaires, making it challenging to establish causality and investigate variables with complex issues of approach, such as drug use, which may influence the authenticity of responses. Secondly, the overall study period was relatively short, considering the ongoing pandemic scenario. However, it is worth noting that this study represents the first screening of asymptomatic indigenous individuals in a Brazilian peri urban area during the pre-vaccination period, providing an essential epidemiological perspective on the spread of SARS-CoV-2 in this population.

## Conclusion

Our study findings showed high seropositivity rate among the asymptomatic indigenous population of Midwest Brazil. This result demonstrates the impact of SARS-CoV-2 on indigenous people living in Brazilian peri urban areas, highlighting worrying scenario. Therefore, enhancing health policies for indigenous communities residing in these regions is crucial, to improve the health of these it.

## Supporting information

S1 Table Contingency table of serological results and concordance ratio between ELISA and rapid test. (DOCX)

S1 File (PDF)

S2 File (PDF)
