# Supplementary material for: Seroprevalence Of SARS-COV-2 infection in asymptomatic indigenous from the largest Brazilian periurban area
Source: PLoS One. 2023 Dec 22;18(12):e0295211. doi: 10.1371/journal.pone.0295211 (PMC10745159; doi:10.1371/journal.pone.0295211)
Supplement: S1 Table — (DOCX) [file pone.0295211.s001.docx]

**Supplementary Table 1.** Contingency table of serological results and concordance ratio between ELISA and Rapid Test.

|  | **Elisa + (%)** | **Elisa - (%)** | **CR** | **Total (%)** |
| --- | --- | --- | --- | --- |
| RT + | 252 (50.8) | 64 (12.9) |  | 316 (63.7) |
| RT - | 10 (2) | 170 (34.3) |  | 180 (36.3) |
| CR |  |  | 422 (85,1) |  |
| Total | 262 (52.8) | 234 (47.2) |  | 496 (100) |

RT: Rapid Test; CR: Concordance Ratio. Rapid Test +: positive results; Rapid Test -: negative results; ELISA +: positive results; ELISA -: negative results; Concordant results, positive and negative, is A and D cells. Discordant results are in B and C cells.
