# Supplementary material for: Seroprevalence Of SARS-COV-2 infection in asymptomatic indigenous from the largest Brazilian periurban area
Source: PLoS One. 2023 Dec 22;18(12):e0295211. doi: 10.1371/journal.pone.0295211 (PMC10745159; doi:10.1371/journal.pone.0295211)
Supplement: S1 File — (PDF) [file pone.0295211.s002.pdf]

UNIVERSIDADE FEDERAL DA GRANDE DOURADOS
Faculdade de Ciências Biológicas e Ambientais
Rodovia Dourados/Itahum, 79.804-970, Dourados, MS, Brazil
Phone: +55 67 34102225, FAX +55 67 34102190
Email: simonesimionatto@ufgd.edu.br

Statement of Ethical Principles - Research with Human Subjects

01st August 2023

All experiments and methods were conducted in accordance with the relevant guidelines and regulations, following the guidelines established by the Brazilian Research Ethics Committee (CONEP), protocol number 4.502.250.

Any and all documents that may be necessary will be presented according to previous request.

Yours Sincerely,

Simone Simionatto, PhD
Laboratório de Pesquisa em Ciências da Saúde,
Universidade Federal da Grande Dourados.
CEP: 79804-970, Dourados - MS, Brazil
Phone: + 55 (67) 3410-2225
Email: simonesimionatto@ufgd.edu.br

UNIVERSIDADE FEDERAL DA GRANDE DOURADOS
Faculdade de Ciências Biológicas e Ambientais
Rodovia Dourados/Itahum, 79.804-970, Dourados, MS, Brazil
Phone: +55 67 34102225, FAX +55 67 34102190
Email: simonesimionatto@ufgd.edu.br

Statement of experimental protocols - Brazilian Committee for Research Ethics (CONEP)

01st August 2023

All experiments were conducted in accordance with the relevant guidelines and regulations, following the guidelines established by the Brazilian Committee for Research Ethics (CONEP), in accordance with the duties defined in the National Health Council (CNS) Resolution No. 466 of 2012 and the Operational Rule No. 001 of 2013 of the same council, research protocoled under number 4,502,250.

Any and all documents that may be necessary will be presented according to previous request.

Yours Sincerely,

Simone Simionatto

Simone Simionatto, PhD
Laboratório de Pesquisa em Ciências da Saúde,
Universidade Federal da Grande Dourados.
CEP: 79804-970, Dourados - MS, Brazil
Phone: + 55 (67) 3410-2225
Email: simonesimionatto@ufgd.edu.br

OPINION ESTABLISHED BY CONEP

AMENDMENT DATA

Search Title: Epidemiology and evolution of SARS-CoV-2 infection in the indigenous population of Mato Grosso do Sul

Researcher: Simone Simionatto

Thematic Area: Studies with indigenous populations;

Version: 6

CAAE: 38981720.5.1001.5160

Bidder Institution: FUNDACAO FEDERAL UNIVERSITY OF GRANDE DOURADOS

Main Sponsor: CONS NAC DE DESENVOLVIMENTO CIENTIFICO E TECNOLOGICO

OPINION DATA

Opinion number: 4,847,966

Project Presentation: The information listed in the "Project Presentation", "Research Purpose" and "Risks and Benefits Assessment" fields were taken from the Basic Research Information file (PB_INFORMAÇÕES_BÁSICAS_1777038_E3.pdf of 06/17/2021).

INTRODUCTION

The Indigenous Health Care Subsystem (SASISUS) was instituted by Law No. 9,836 of 1999 to implement a model of differentiated care for the indigenous population, which requires, in addition to very different logistics, an approach to cultural aspects that need to be incorporated into care for health of this population. The National Health Care Policy for Indigenous Peoples (PNASPI) was enacted in 2002 and led to the formulation of a work model based on 34 Special Indigenous Health Districts (DSEI) throughout the Brazilian territory, which may cover more than one municipality or state. . In addition to the DSEI, the management staff of SASISUS comprises the Base Hub and the Basic Indigenous Health Units (UBSI), with the Special Secretariat for Indigenous Health (SESAI) being the area of the Ministry of Health responsible for coordinating the PNASPI (BRASIL, 2019) .The actions of primary care for indigenous health and basic sanitation are carried out in villages and on indigenous lands, through the organization of a comprehensive care network made up of Multidisciplinary Teams of Indigenous Health (EMSI) made up of doctors, nurses, psychologists, nutritionists , dentists and other health professionals who work in conjunction with

other levels of the Unified Health System (SUS), for specialized care of medium and high complexity (BRASIL, 2019). The population assisted by SASISUS in 2018, according to the Indigenous Health Care Information System (SIASI), was 737,262 indigenous people distributed in 24 UF, 494 Brazilian municipalities, 4580 villages and 303 ethnic groups (SIASI/SESAI, 2014). social vulnerability, historically associated with gender inequality, poverty and social conflicts, are frequently present in the field of communicable diseases and thus justify the development of approaches that relate the socioeconomic and demographic factors that favor their occurrence in the indigenous population. In addition, social, biological, cultural and behavioral aspects are important aspects in the transmissibility of infectious diseases, influencing their occurrence. These aspects reinforce the importance of approaching vulnerable and poorly assisted populations such as the indigenous population (PINTO et al., 2014). The new corona virus (SARS-CoV-2), which causes COVID-19, which emerged in Wuhan, China, at the end of December 2019, quickly spread to other countries (VELAVAN; MEYER, 2020), was declared by the Organization World Health Organization (WHO), on January 30, 2020, a Public Health Emergency of International Concern (PHEIC) and on March 23, 2020 as a pandemic (CRODA et al., 2020). Efforts to contain the virus are ongoing. However, given the many uncertainties regarding the transmissibility and virulence of this pathogen, the effectiveness of these efforts is unknown (LI et al., 2020)). The fraction of undocumented but infectious cases is a critical epidemiological characteristic that modulates the pandemic potential of an emerging respiratory virus (CHAN et al., 2020; WU et al., 2020). These undocumented infections often have mild, limited or no symptoms and therefore go unnoticed and, depending on their contagiousness and numbers, can expose a much larger portion of the population to the virus than would normally occur (LI et al., 2020). Indigenous and non-indigenous peoples are immunologically susceptible to viruses that have never circulated before, such as SARS-CoV-2. Fears of the devastating impact this virus could have on indigenous communities in South America grew after a teenager from the Yanomami people of Brazil died from the infection in the Amazon (PHILLIPS, 2020). Studies in various parts of the world and in Brazil attest that indigenous people are more vulnerable to epidemics (ZAVALETA, 2020), which may be associated with worse social, economic and health conditions for the rest of the population, which amplifies the potential for spread of diseases. Particular conditions affect these populations, such as the difficulty of accessing health services, whether due to geographical distance, or the unavailability or insufficiency of health teams. To track sources of infection and protect these vulnerable people, we need to

related to the circulation of the virus in these communities. COVID19 control actions require epidemiological studies and population surveillance systems that promote the production of quality evidence on the effectiveness of individual intervention and in the territories, mainly because it is a serious disease with a high transmissibility. In addition, with the possibility of asymptomatic occurrence, the scenario of COVID19 can be worsened in places where there are no timely detection strategies (LI et al., 2020).

HYPOTHESIS

Considering that the State of Mato Grosso do Sul has the second largest indigenous population in the country, carrying out this study will provide knowledge on the transmission of SARS-CoV-2, enabling a better understanding of the transmission profile of this virus in this population, the seroprevalence, as well as prognostic factors for severity and death.

METHODOLOGY

Data collectionThe collection of biological material for the RT-PCR tests follows the flow already established in the basic indigenous health units (UBSI), using nasal swabs. The purpose of this study is to analyze symptomatic individuals for COVID-19 through the analysis of nasal swabs for molecular biology tests, using polymerase chain reaction (PCR) and/or sample of blood for tests that detect IgM/IgG antibodies against SARS-CoV-2, considered a quick test (point of care). From the patients tested positive, after the TCLE, a blood sample will be collected for serological and immunophenotypic assays, within 15 days after the onset of symptoms and/or vaccination. From patients tested positively, 4.5 ml of blood will be collected for serological and immunophenotypic assays. Positive patients will be divided into three groups defined according to mild, moderate and severe clinical conditions, in addition to a control group of seronegative and asymptomatic individuals. Clinical, immunological and outcome conditions of the cases will be monitored. Monitoring over 24 months on the permanence and concentration of antibodies against SARS-CoV-2 will be performed by ELISA with blood collection at 12 and 24 months after diagnosis and/or vaccination. To determine the percentage of asymptomatic or subclinical infections with SARS-CoV-2, 1,000 asymptomatic patients will be recruited. Residences will be drawn randomly and serological testing will be performed to detect IgM/IgG antibodies against SARS-CoV-2 (considered a rapid test). Through the project, a nurse residing in the Dourados indigenous reserve will be hired

for collections,

which will be available to the DSEI-Polo base in Dourados to support health surveillance actions and will follow the logistics established by the Polo team. The displacement of health professionals linked to the research to the territory will take place with resources from this project. Blood collections will be performed weekly or when there are positive cases for SARSCoV-2. Characterization of cellular immune response and seroconversion of patients. Cells involved in virus-specific immune responses, concentrations of immunoglobulins M (IgM) and G (IgG) will be characterized by immunophenotyping using the flow cytometry technique. The presentation profile of the immunological responses will be confronted with the clinical conditions and ancestry markers of the evaluated patients.
expression of ancestry markers. A panel with 61 informative markers of ancestry, already validated for the Brazilian population, will be analyzed. Polymorphisms will be analyzed using multiplex PCR and capillary electrophoresis in an ABI3130xl automatic sequencer.Sequencing of circulating SARS-Cov2 strains in the indigenous population -PCR Kit and submitted to sequencing using the Illumina platform. The sequencing result will be analyzed using bioinformatics tools that will allow identifying the genetic characteristics of SARS-CoV-2.

INCLUSION CRITERIA

- Indigenous patients with a clinical picture of the flu syndrome, identified and notified by the DSEI/MS, will be included in the study.
- Indigenous patients who sign the Term of Free and Informed Consent (TCLE) or Term of Free and Informed Assent (TALE).
- Indigenous patients vaccinated against SARS-CoV-2.

EXCLUSION CRITERIA

- Non-indigenous patients without clinical symptoms of flu syndrome.
- Indigenous patients who do not sign the TCLE.

Research Purpose:

PRIMARY OBJECTIVE(S)

Evaluate the evolution of SARS-CoV-2 infection in the indigenous population of Mato Grosso do Sul and carry out a molecular epidemiology study of this virus.

SECONDARY OBJECTIVE(S)

- Determine the number of patients with flu syndrome during the study period;
- Determine the number of infections per epidemiological week;
- Evaluate clinical manifestations in patients with a confirmed diagnosis;
- Describe the dynamics of SARS-CoV-2 transmission between contacts in different ethnic groups;
- Determine the percentage of asymptomatic infections with SARS-CoV-2 and risk factors associated with COVID infection in the indigenous population;
- Identify the percentage of infections with SARS-CoV-2 in indigenous health professionals;
- Provide estimates of the percentage of infections that allow accurate calculations of the lethality of the disease;- Associate time of onset of seroconversion and change of immunoglobulin class isotypes to the prognostic classification;

- Evaluate by ELISA over 24 months the permanence and concentration of antibodies in individuals exposed to SARS-CoV-2 and/or vaccination;
- Characterize cellular and molecular components for qualitative determination of the immune response, associated with ancestry markers.
- Determine the genetic characteristics of SARS-CoV-2 circulating in the indigenous population;
- Evaluate the surveillance measures regarding the contingency of the disease in the indigenous territory;
- Support the actions proposed by health services regarding health education and actions based on scientific evidence;

- Contribute to the epidemiological surveillance system in the description of the clinical-epidemiological profile of infections caused by SARS-CoV-2 in the indigenous population of the state of Mato Grosso do Sul.

Assessment of Risks and Benefits:

SCRATCHS

The risks are restricted to those related to some type of discomfort during the collection of material, where patients may feel a small sensation of discomfort at the site of the needle prick, which can be minimized by being collected by professionals duly trained for collection. of blood and performed with small gauge needles. If there is any discomfort, patients will receive care from the health team. This collection will be carried out by indigenous health professionals, according to the logistics established by the health services. Participation in the research may cause negative memories, or even possible risks related to the vulnerability of indigenous communities with the knowledge of the characteristics of the immune response generated by the disease, and with that psychological damage. The team

is trained to minimize such risks, and if it happens, we guarantee full and free assistance for damages caused as a result of the study. The participant is guaranteed by the resolution of the National Health Council no. 466 of 2012, the right to seek compensation and reimbursement resulting from the research if he feels injured or harmed. The main researcher is responsible for future indemnities and reimbursements that may occur as a result of this research, as provided for in Brazilian legislation.

BENEFITS

As benefits, we hope this research can help local health agencies and DSEI to develop contextually appropriate interventions that should be effective in helping this vulnerable population. In addition to expanding studies on the subject in order to support interventions aimed at improving the quality of life of indigenous populations.

Comments and Considerations about the Research:

Amendment E3:

According to what is informed in the file “Amendation3_CONEP.pdf”, the main reason for the amendment is: “This amendment aims to sequence SARS-CoV-2 samples from indigenous patients stored at LACEN/MS.”.

Considerations about the Mandatory Presentation Terms: See field "Conclusions or Pending Issues and List of Inadequacies".

Conclusions or Pending Issues and List of Inadequacies: No ethical obstacles were identified in this amendment.

Final Considerations at the discretion of CONEP: In view of the above, the National Research Ethics Commission - Conep, in accordance with the attributions defined in CNS Resolution No. of the proposed amendment to the research project.

Status: Amendment approved.

This opinion was prepared based on the documents listed below:

Document Type	File	Post	Author	Situation

Basic information from the project	PB_INFORMAÇÕES_BASIC_1777038_E3.pdf	06/17/2021 16:29:53		Accepted
Others	Amendment3_CONEP.pdf	06/17/2021 16:23:33	Simone Simionatto	Accepted
Others	ANUENCIA_SES_amostras_Lacen.pdf	06/17/2021 16:22:44	Simone Simionatto	Accepted
Previous Opinion	OPINION_E1_APROVADO.pdf	03/17/2021 17:58:36	Simone Simionatto	Accepted
Detailed project / Brochure Investigator	projectNoHighlightEmenta2.docx	03/17/2021 17:55:04	Simone Simionatto	Accepted
Brochure Search	projectComDestaqueEmenta2.docx	03/17/2021 17:54:40	Simone Simionatto	Accepted
Others	AMENDMENT2_CONEP.pdf	03/17/2021 17:53:59	Simone Simionatto	Accepted
Others	TCLE_Responsavel.pdf	01/26/2021 20:40:00	Simone Simionatto	Accepted
TCLE / Terms of assent / Justification of Absence	TCLE.pdf	01/26/2021 20:38:05	Simone Simionatto	Accepted
Previous Opinion	OPINION_APROVADO.pdf	01/21/2021 19:56:14	Simone Simionatto	Accepted
Others	TALE_13a18_anos.pdf	01/21/2021 19:54:27	Simone Simionatto	Accepted
Others	TALE_06.pdf	01/21/2021 19:54:06	Simone Simionatto	Accepted
Others	declarationBiorrepositorioUFGD_UFBa.pdf	10/04/2020 22:12:07	Simone Simionatto	Accepted
Others	ResolutionFCBAProjectCOVIDCNPq.pdf	10/04/2020 21:23:56	Simone Simionatto	Accepted
Others	CVSimone.pdf	10/04/2020 21:23:01	Simone Simionatto	Accepted
Others	questionnaire.pdf	10/04/2020 21:22:01	Simone Simionatto	Accepted
Others	OficioSES_autorizando.pdf	10/04/2020 21:21:28	Simone Simionatto	Accepted
Others	oficioFUNAI.pdf	10/04/2020 21:20:52	Simone Simionatto	Accepted
Others	ProtocolFunai.pdf	10/04/2020 21:20:27	Simone Simionatto	Accepted
Others	LiderancaJaguapiru.pdf	10/04/2020 21:19:36	Simone Simionatto	Accepted
Others	LiderancaBororo.pdf	10/04/2020 21:19:10	Simone Simionatto	Accepted
Others	ConselhoLocalIndigena.pdf	10/04/2020 21:18:28	Simone Simionatto	Accepted

Others	AtaCONDIS4.pdf	10/04/2020 21:17:19	Simone Simionatto	Accepted
Others	AtaCONDISI3.pdf	10/04/2020 21:16:59	Simone Simionatto	Accepted
Others	AtaCONDISI2.pdf	10/04/2020 21:16:35	Simone Simionatto	Accepted
Others	AtaCONDISI.pdf	10/04/2020 21:15:49	Simone Simionatto	Accepted
Others	Consent CONDISI.pdf	10/04/2020 21:15:15	Simone Simionatto	Accepted
Others	Signed Agreement.pdf	10/04/2020 21:14:43	Simone Simionatto	Accepted
Declaration of agreement	ConsentDSEI.pdf	10/04/2020 21:13:16	Simone Simionatto	Accepted
Budget	budgetCNPq.docx	10/04/2020 21:11:30	Simone Simionatto	Accepted
Declaration of Sponsor	grantCNPq.pdf	10/04/2020 21:11:11	Simone Simionatto	Accepted
Declaration of Researchers	declaracaocoordeandor.pdf	10/04/2020 21:10:45	Simone Simionatto	Accepted
Declaration of Material Handling biological / Biorepository / Biobank	RegulationBiorrepositoryUFGD.pdf	10/04/2020 21:09:47	Simone Simionatto	Accepted
Declaration of Institution and Infrastructure	AnnuenciaPROPP.pdf	10/04/2020 21:09:13	Simone Simionatto	Accepted
Timeline	Schedule.docx	10/04/2020 21:08:53	Simone Simionatto	Accepted
Others	Fabiano.pdf	10/04/2020 21:08:38	Simone Simionatto	Accepted
Title Page	FolhaDeRostoCNPqSeptember2020Fabiano.pdf	10/04/2020 20:58:06	Simone Simionatto	Accepted

Status of the Opinion:

Approved

BRASILIA, July 14, 2021

Signed by:
Jorge Alves de Almeida Venancio
(Coordinator)

Term of Free and Informed Consent – TCLE

You are being invited to participate, as a volunteer, in the research entitled "Epidemiology and evolution of SARS-CoV-2 infection in the indigenous population of Mato Grosso do Sul." My name is Simone Simionatto and I am the researcher responsible for this study. I clarify that if you decide not to participate, you will not be penalized in any way or prevented from participating in other research. But if you agree to participate, your questions about the research can be clarified via e-mail (simonesimionatto@ufgd.edu.br) or by phone number: (67) 3410-2225. Doubts about your rights as a participant in this research, you can also clarify with the Ethics Committee on Research with Human Beings (CEP) of UFGD, by phone (67) 3410-2853 or by e-mail cep@ufgd.edu.br. The CEP of the Federal University of Grande Dourados (UFGD) is an interdisciplinary and independent collegiate, advisory, deliberative and educational and aims to defend the interests of research subjects in their integrity, dignity, safety and well-being, contributing to the development of research within ethical standards. . Open from Monday to Thursday, from 8am to 11am and from 2pm to 5pm, and on Fridays from 8am to 11am, at Rua João Rosa Góes, 1761 – Vila Progresso, Dourados/MS. If you wish, you can also contact the National Research Ethics Commission (Conep), SRTV 701, Via W 5 Norte, lot D - PO 700 Building, 3rd floor – North Wing CEP: 70719-040, Brasília/DF).
We are carrying out this research to evaluate the evolution of the disease by the new coronavirus in the indigenous population of Mato Grosso do Sul and thus discover new ways to control COVID-19 in the villages. After receiving the following clarifications and information, you will have some time to think and decide whether you want to participate in this research. If you accept, initial all pages and sign at the end of this document, which is printed in duplicate, one of which is yours and the other will be filed with me. If you sign this term, you authorize the collection, deposit, storage and use of human biological material, which will be used for research as provided in this term and in the project. These samples will be stored at the Health Sciences Research Laboratory (LPCS) at the Federal University of Grande Dourados for a period of ten years and subsequently destroyed. Your biological samples will only be used as described in this term and in the project submitted to CEP and CONEP. If your biological sample is used for another purpose not described in this term, we will contact you for a new authorization and collection of signatures in a new term that will explain the future procedures that will be carried out with your sample and your data not foreseen in this project . This will only be done after future studies are approved by the CEP/CONEP system.

Your participation in this research will be in 3 stages:

In the first step, you will be invited to participate in the research and provide samples of blood and/or nasal discharge for laboratory tests. If you agree to participate in the study, some questions will be asked by duly trained professionals with an average duration of 3 to 5 minutes. The material collected from the nose and the blood will be used to perform tests for the diagnosis of COVID-19 and monitor the evolution of the disease and/or vaccination. If the sample collected from the nose is positive, this same sample will be used to identify the coronavirus and find out whether the virus that caused the disease is identical to the virus present in other patients. **In the second and third stages**, 4.5 mL of blood will be collected 12 and 24 months after the diagnosis of COVID-19 and/or vaccination. This exam will monitor the permanence and concentration of cells in the defense system against the coronavirus. At any time, you may withdraw the consent for the custody and use of the samples stored in our laboratory by means of a written and signed statement. After withdrawal of consent these will be returned to you. All of your collected information, both from the interview and from the exams performed, will be stored in a confidential database. Your name will be associated with a code, which only the main researcher knows, to ensure that your participation is anonymous. The results of this study may be presented at scientific meetings or published in scientific journals. However, your identity will not be disclosed. The risks that the research may cause you are related to the collection of material from the nose and blood, which can generally cause rapid discomfort at the site, but which will be minimized when conducted by experienced professionals. Conducting the interview may raise negative memories, or even possible risks related to the vulnerability of indigenous communities with knowledge of the characteristics of the immune response generated by the disease, and with that psychological damage. If you feel unwell, you will receive care from the health team that will be assisting you. Our team is trained to minimize such risks, and if it happens, we guarantee full and free assistance for damages caused as a result of the study. You are guaranteed by Resolution of the National Health Council No. 466 of 2012, the right to seek compensation and reimbursement arising from the research if you feel injured or harmed. The main researcher is responsible for future indemnities and reimbursements that may occur as a result of this research, as provided for in Brazilian legislation.

Participant Name: ______________________________ Signature: ______________________
Responsible researcher: Dr. Simone Simionatto Signature:______________________

As benefits, we hope this research can contribute to local health agencies and DSEI to develop appropriate and effective interventions to help this population. In addition to expanding studies on the disease in order to support interventions aimed at improving the quality of life of indigenous populations. We emphasize that there will be no costs for you. You may refuse to participate or even leave the research at any time without prejudice, sanctions or embarrassment. You will not receive any form of payment for participating. Participation in this work will not influence your treatment and care provided to you by the health team. You may request information about the survey at any time and even after the survey has ended.
Your exam results will be delivered to you within 72 hours. All people diagnosed will be guaranteed access to the treatment recommended by the Ministry of Health, who will be referred for treatment at the indigenous health services. In this work no new drug will be tested for the treatment.
And finally, we guarantee you:
1. Preserve the secrecy of all information made available;
2. Make use of the information obtained in the research in presentation and/or publications exclusively for the purpose foreseen in the protocol and according to the consent that you are signing;
3. Not to divulge or transfer to third parties the information collected in the collected material;
4. I declare that the results will be used only for this research;
5. Not to explore, reproduce or use the information for any purpose other than the specific one in the previous items;
6. The survey results will be presented to the leaders of the surveyed communities in meetings scheduled together with the DSEI/MS team;

I declare that I understand the objectives, risks and benefits of my participation in the research and I agree to participate.

( ) YES, I want to participate in the research.

Participant Name: _________________________________ Signature: ___________________

or fingerprint:

Thumbprint:

Responsible researcher: Dr. Simone Simionatto Signature:____________________________
Date: _________________

Name and telephone number of the responsible researcher:
Dr. Simone Simionatto
Phone: (67) 3410-2225
Federal University of Grande Dourados – FCBA Block
Dourados-Itahum Highway km 12
University City, no.

Committee for Ethics in Research with Human Beings (CEP)
Address: Rua João Rosa Góes, 1761 – Vila Progresso, Dourados-MS.
CEP: 7.9825-070, E-mail: cep@ufgd.edu.br, Telephone: (67) 3410-2853
Opening hours:
Monday to Thursday from 08:00 to 11:00 and from 14:00 to 17:00 and on Friday from 08:00 to 11:00.
